# Precipitants of 5-Fluorouracil in Trabeculectomy Bleb Management: A Comparative Laboratory Study

**DOI:** 10.5005/jp-journals-10008-1247

**Published:** 2018-08-01

**Authors:** Karl J Mercieca, Cecilia H Fenerty, Laura R Steeples, Brett Drury, Archana Bhargava

**Affiliations:** 1 Consultant and Surgeon, Surgeon and Glaucoma Fellow, Manchester Royal Eye Hospital, Oxford Road, Manchester, UK; 2 Consultant and Surgeon, Aintree University Hospitals NHS Foundation Trust, Longmoor Lane, Liverpool, UK

**Keywords:** Anti-metabolite, Bleb management, 5-FU, Needling, Trabeculectomy.

## Abstract

**Aim:**

Concerns regarding 5-Fluorouracil (5-FU) corneal toxicity have resulted in various ways of reducing its corneal exposure during post-operative trabeculectomy bleb manipulation. This study investigates the properties of various topical agents used to induce the precipitation of this compound.

**Materials and methods:**

This is a double-blind, descriptive, laboratory study comparing five different potential precipitants of 5-FU (proxymetacaine (proxy), oxybuprocaine (oxy), ametho-caine (ameth), fluorescein (flor), proxymetacaine + fluorescein (proxy-flor) to a control group (normal saline). A 0.01 mL of each anonymized agent was applied next to a clear round comparison marker in a transparent sterile container set on a dark background. 5-FU (0.01 mL of 50 mg/mL) was subsequently applied to each agent. The induced changes in transparency were imaged and compared to the transparency of the central marker. For each application, pH changes were also noted.

**Results:**

Proxy, flor and proxy + flor did not result in any discernible 5-FU precipitation. Oxy resulted in a moderate visible change, and ameth produced very significant precipitation. Application of proxy, oxy, ameth, and proxy + flor resulted in the neutralization of the 5-FU’s alkaline pH.

**Conclusion:**

We propose using a cotton-tipped bud dipped in ameth applied at the injection site as the recommended method to avoid 5-FU corneal exposure in these cases.

**Clinical significance:**

Practitioners should be aware of the differences in precipitation of 5-FU by different types of topical anesthetics and modify techniques of anesthesia and 5-FU administration accordingly.

**How to cite this article:** Mercieca KJ, Fenerty CH, Steeples LR, Drury B, Bhargava A. Precipitants of 5-Fluorouracil in Trabeculectomy Bleb Management : A Comparative Laboratory StudyJ Curr Glaucoma Pract 2018;12(2):64-66.

## INTRODUCTION

The last twenty years have witnessed an evolution in trabeculectomy techniques.^1,2^ The application of intra-and post-operative anti-metabolites for influencing the wound healing process has led to significantly enhanced success rates of this procedure.^3-6^ 5-FU is a pyrimidine analog commonly injected into the subconjunctival space to prevent scarring during post-trabeculectomy bleb management. Concerns regarding 5-FU corneal toxicity have resulted in various ways of reducing its corneal exposure including the modification of injection administration, ocular surface irrigation, and application of topical agents to induce 5-FU precipitation.^7,8^

Amethocaine (tetracaine) has classically been used to precipitate 5-FU after injection either to prevent reflux exposure to the cornea or by highlighting reflux to identify those corneas at higher risk and need of more copious irrigation.^7,8^ However, some clinicians use different topical agents both before and following 5-FU injection including fluorescein, proxymetacaine, benoxinate (oxybuprocaine) and amethocaine (tetracaine).

This study explores the different precipitation properties of commonly used topical agents when added to 5-FU and the effect on pH brought about by these interactions. The results are used to propose a standardized way of administering 5-FU post-trabeculectomy in a safe and repeatable manner.

## MATERIALS AND METHODS

This is a double-masked, descriptive, laboratory study which compares five different potential 5-FU precipitants to a control group of normal saline. The chosen agents were preservative free proxymetacaine 0.5%, oxybu-procaine 0.4%, amethocaine 0.5%, fluorescein 0.25% + proxymetacaine 0.5% + fluorescein 0.25% (Bausch and Lomb, Minims®, New York, USA). Sterile saline eye drops (Bausch and Lomb, Minims®, New York, USA) were selected as a neutral control.

A metallic trolley was set up in a clean room with six sterile transparent containers positioned on a black background to increase contrast. Each container was designated a letter (A to F), and 0.01 mL of 5-FU (from a 50 mg/mL vial) (Accord Healthcare Ltd, Harrow, England) was applied with an insulin syringe adjacent to a clear circular comparison marker in the center of the container.

This step was performed by investigator 1 (KM) in the absence of investigator 2 (LS). Investigator 2 (LS) subsequently applied 0.01mL of each potential precipitant or normal control to the 5-FU droplets using separate insulin syringes each of which was anonymized and randomly allocated to a 5-FU group (A-F).

Alteration in transparency was chosen as a surrogate of precipitation, and the resultant changes were photographed and compared to the central marker. The pH of the 5-FU and of each potential precipitant was measured using Universal Indicator pH-indicator paper (Merck, New Jersey, USA) by KM before application. The pH was re-measured by LS following the mixing of agents with 5-FU to ensure the masking of each group.

The photos were examined by investigator 2 (LS) and scores of 1-5 given: 1-no obvious precipitation, 2-mild visible precipitation, 3-intermediate visible precipitation, 4-very significant precipitation, 5-classification not possible. The groups were then unmasked and the scores applied to each group. The change in pH was then annotated for every reaction between agents.

## RESULTS

**Fig. 1:**
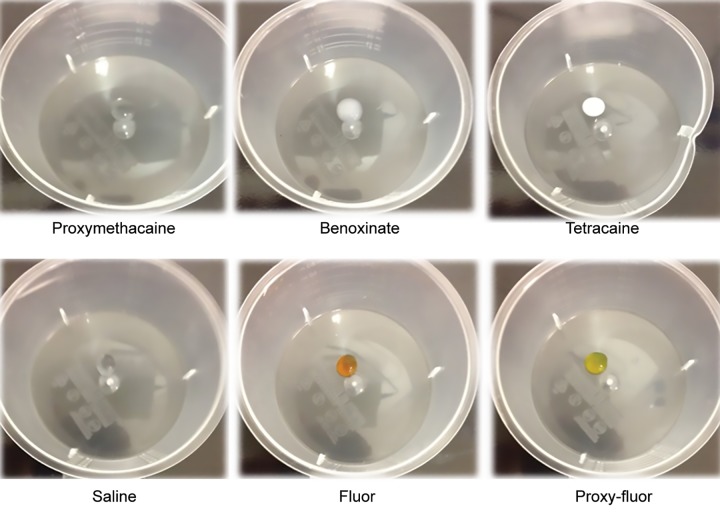
Photographs of all six unmasked containers after application of 5-FU. Change in transparency (opacification) was compared to the central clear markers (white lines) and scored from 1 to 5 as described in text.

Proxymetacaine, fluorescein, and proxymetacaine + fluorescein, did not result in any discernible 5-FU precipitation and were assigned a score of 1. Oxybuprocaine (benoxinate) induced a moderate visible change (score 3) whilst amethocaine (tetracaine) produced very significant opacification (score 4) (Fig. 1).

5-FU was found to have a pH of 8.5 before mixing with a topical agent. Application of proxymetacaine, oxybuprocaine, amethocaine and proxymetacaine + fluorescein (all pH 5.5) resulted in the neutralization of the 5-FU’s alkaline pH whereas, as expected, saline and flor (pH 7) both maintained the alkaline pH (8.5) after application.

## DISCUSSION

Precipitation is the creation of a solid within a solution during a chemical reaction. Without sufficient settling to bring the solid particles together, the precipitate remains in suspension, resulting in the white color changes observed. Benoxinate and amethocaine were the only topical agents which produced visible precipitation of 5-FU with the latter exhibiting a greater propensity to do so. This seems to justify the choice of amethocaine by many clinicians for precipitating 5-FU following injection.

Precipitation does not necessarily result in deactiva-tion of the drug as molecules can still be present in any residual solution and 5-FU may remain active in the solid state. This study did not look into the chemical properties of precipitated 5-FU but instead aimed to define which agents did or did not cause it to precipitate out of solution. This is important when looking at the concept behind precipitating 5-FU to prevent corneal toxicity. Amethocaine application over the entire cornea in an attempt to neutralize the 5-FU spillover does not seem justified in this context, even if this is eventually washed off with saline. The addition of amethocaine specifically to test for drug reflux seems more logical and its use to tamponade the needle entry site thus precipitating 5-FU and blocking corneal exposure even more so. In our practice, we find that application of amethocaine on a cotton-tipped bud is the most effective way of achieving this. Our preferred technique is following injection of 5-FU, the needle is gently withdrawn back to the entry site and paused just before exiting the subconjunctival space. This step aims to prevent reflux. Then the syringe is immediately replaced with a cotton bud previously soaked in amethocaine. This step allows the application of mechanical pressure to the entry site and provides a physical barrier to 5-FU reflux while capturing any excess by precipitation. The pushing action of the tip also helps the 5-FU spread more diffusely underneath the bleb. If precipitated 5-FU does reach the corneal surface, it is washed copiously with normal saline.

This study also confirms the pH neutralizing effect that all acidic topical anesthetics have on alkaline 5-FU. One must bear in mind however that pH values can vary (depending on solubility properties) and that all anesthetic agents have different detergent properties which may affect absorption into corneal and conjunctival tissues.^9^ Topical amethocaine, for example, can produce temporary superficial punctate keratitis and corneal edema which could significantly increase 5-FU exposure to corneal tissue and/or contribute to post-injection corneal changes.^10^

## CONCLUSION

We suggest that amethocaine (tetracaine) should be the topical agent of choice for 5-FU precipitation when this is used to prevent corneal toxicity after sub-conjunctival bleb injection. We recommend using a cotton-tipped bud dipped in ameth applied to the injection entry point as a safe and standardized technique to avoid 5-FU corneal exposure in these situations.

## CLINICAL SIGNIFICANCE

Practitioners should be aware of the differences in precipitation of 5-FU by different types of topical anesthetics and modify techniques of anesthetics and 5-FU administration accordingly.
